# Development of Fluorescence-Based Method for Dopamine Determination Using o-Phthaldialdehyde and 3-Mercaptopropyltriethoxysilane

**DOI:** 10.3390/s25061729

**Published:** 2025-03-11

**Authors:** Valeriia Sliesarenko, Marijana Krstić, Urban Bren, Aleksandra Lobnik

**Affiliations:** 1 Institute for Environmental Protection and Sensors, IOS Ltd., 7 Beloruska Str., SI-2000 Maribor, Slovenia; 2 Faculty of Chemistry and Chemical Engineering, University of Maribor, 17 Smetanova Str., SI-2000 Maribor, Sloveniaurban.bren@um.si (U.B.); 3 Faculty of Mathematics, Natural Sciences and Information Technologies, University of Primorska, 8 Glagoljaška Str., SI-6000 Koper, Slovenia; 4 Faculty of Mechanical Engineering, University of Maribor, 17 Smetanova Str., SI-2000 Maribor, Slovenia; aleksandra.lobnik@um.si

**Keywords:** fluorometric method, dopamine, o-phthalaldehyde

## Abstract

Nanomaterials and sensors play an important role in modern technologies, including medical diagnostics and biochemical research. This work presents the possibility of using o-Phthaldialdehyde (OPA) in combination with 3-mercaptopropyltriethoxysilane (MPTES) to develop a dopamine-responsive sensor. During the experiment, these materials were used at different pH and ratios to determine the optimal parameters for obtaining high fluorescence intensity of the reaction product. The data obtained demonstrate a linear relationship between the fluorescence response (λ_ex_/λ_em_ = 340/460 nm) of OPA/MPTES and dopamine concentration in the range of 0.1–3.0 µM at a pH of 8, and the detection limit was 8.7 nM. The obtained results confirm the potential of OPA/MPTES as a sensing component for the detection of dopamine.

## 1. Introduction

Dopamine (DA) is an important hormone and neurotransmitter, and its levels in human fluids can indicate stress, depression, and various mental disorders [1,2]. Food products, as well as medications, can affect its level in the human body. Despite the significance of dopamine measurements in fields like neuroscience, diagnostics, and pharmacology [3,4], the range of accessible analytical methods still remains limited. The most commonly used approaches involve either expensive and labor-intensive chromatographic techniques or optical methods with relatively low sensitivity and high detection limits [5,6].

Advanced methods of dopamine detection include both electrochemical and optical approaches, specifically the use of gold nanoclusters [7] and other metals [8], metal–organic frameworks (MOFs) [9], and graphene [8,10], often in combination with aptamers [10] or antibodies [11]. These technologies demonstrate high selectivity and sensitivity and could potentially form the basis for portable sensor systems [12]. However, their widespread practical application is currently limited by complex and labor-intensive manufacturing processes, high costs, and a number of environmental issues [13,14], significantly restricting their availability for routine or mass clinical studies.

**Scheme 1:**
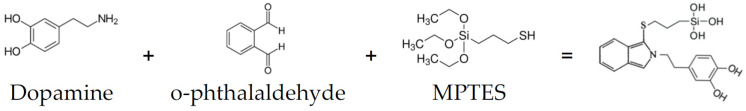
Starting components and presumed product.

At the same time, the niche for simple and accessible analytical solutions for dopamine detection remains underdeveloped. One such approach involves the use of indicators. Previous research has shown that in the presence of dopamine, o-phthaldialdehyde (OPA) produces a weak, rapidly fading fluorescent signal [5]. Additionally, the presence of sulfur-containing nucleophiles can significantly enhance the fluorescence of the resulting products [15]. In our study, we examined the fluorescent response of the reaction between dopamine and OPA in the presence of 3-mercaptopropyltriethoxysilane (MPTES), which acts as an enhancing agent. Scheme 1 presents a simplified schematic of the reaction, showing the starting components and the presumed product.

Table 1 compares various spectrofluorescence methods used for dopamine detection. The fluorescence-based methods in Table 1 require additional steps to modify or interfere with the reaction pathway, such as the incorporation of external agents or stopping the reaction, making the overall process more complex, time-consuming, and labor-intensive. In contrast, the approach proposed in our research is a rapid, one-step method that directly enhances the fluorescence response during the reaction, achieving reliable results in just 10 min without the need for additional modifications.

Additionally, we demonstrated that various parameters of the fluorimeter, such as lamp power and excitation and emission slit widths, significantly influence the detection limits. By optimizing these parameters, we achieved improved sensitivity in dopamine measurements, highlighting the importance of instrumental adjustments in enhancing the performance of fluorescence-based methods.

## 2. Materials and Methods

### 2.1. Chemicals

Phthaldialdehyde–o-Phthalaldehyde (≥99.0% (HPLC)), dopamine hydrochloride (Pharmaceutical Secondary Standard), l-Norepinephrine hydrochloride (≥98.0%), Serotonin hydrochloride (≥98%), (±)-Octopamine hydrochloride, Agmatine sulfate salt (≥98.0% (HPLC), Arginine hydrochloride, l-Glutamine (≥99.0%), l-Alanine, Glycine, l-Glutathione (≥98.0%), Urea, and NaHCO_3_ (99.7%) were purchased from Sigma Aldrich, St. Louis, MO, USA.

Ethanol absolute (99.98%), NaOH (99%), and KH_2_PO_4_ (99%) were obtained from Gram Mol d.o.o., Zagreb, Croatia. Melatonin and l-histidine, both Pharmaceutical Secondary Standards, as well as Na_2_HPO_4_ (ACS, Reag.PhEur), were obtained from Merck KGaA, Darmstadt, Germany.

### 2.2. Buffer Solutions

The buffer solutions were prepared using the following ratios:

pH 6: 100 mL 0.1 M KH_2_PO_4_ and 11.2 mL 0.1 M NaOH;

pH 7: 100 mL 0.1 M KH_2_PO_4_ and 58.2 mL 0.1 M NaOH;

pH 7.5: 100 mL 0.1 M KH_2_PO_4_ and 82.2 mL 0.1 M NaOH;

pH 8: 100 mL 0.1 M KH_2_PO_4_ and 93.4 mL 0.1 M NaOH;

pH 9.6: 100 mL 0.05 M NaHCO_3_ and 10 mL 0.1 M NaOH;

pH 10: 100 mL 0.05 M NaHCO_3_ and 21.4 mL 0.1 M NaOH;

pH 11: 100 mL 0.05 M Na_2_HPO_4_ and 8.2 mL 0.1 M NaOH;

pH 12: 100 mL 0.05 M Na_2_HPO_4_ and 53.8 mL 0.1 M NaOH.

The prepared buffer solutions were stored at 4 °C and utilized within three days to ensure their stability and effectiveness.

### 2.3. Work Solution Composition

Dopamine (5 µM) and OPA (100 µM) with MPTES (200 µM): 0.8 mL of buffer solution; 0.1 mL of OPA solution in EtOH (0.004 M); 0.1 mL of MPTES solution in EtOH (0.008 M); 2.0 mL of distilled water and 1.0 mL of dopamine water solution (conc. 2 × 10^−5^ M).

### 2.4. Instrumentation

Fluorescence spectra were acquired using the Fluorescence Spectrometer LS55 (Perkin Elmer, Waltham, MA, USA). The lamp power (denoted as H for high, M for medium, and L for low) and the widths of the excitation and emission slits were adjusted according to the measured fluorescence intensity. Slit widths of 3 nm, 5 nm, or 10 nm were used for both excitation and emission, and denoted as 3/3, 5/5, or 10/10, respectively. All measurements were performed at room temperature.

### 2.5. Calculations

The calculation of the standard deviation (SD), limit of detection (LOD), and limit of quantitation (LOQ) was carried out as described previously [5]. *SD* was determined using the formula *SD* = √(Σ(*IF* − *MV*)^2^/*n*), where *IF* is each measured fluorescence intensity, and *MV* is the mean value across the n recorded intensities. The *LOD* and *LOQ* were established based on the standard deviation of blank measurements and the slope from the linear calibration plot. Specifically, *LOD* = 3 × *SD*/*k* and *LOQ* = 10 × *SD*/*k*, where *SD* corresponds to the standard deviation derived from five blank samples, and *k* is the slope in the linear equation *IF* = *m* + *k* × [*DA*], with [*DA*] being the dopamine concentration (in mol/L).

## 3. Results and Discussion

### 3.1. Influence of pH on Fluorescence Intensity

**Figure 1:**
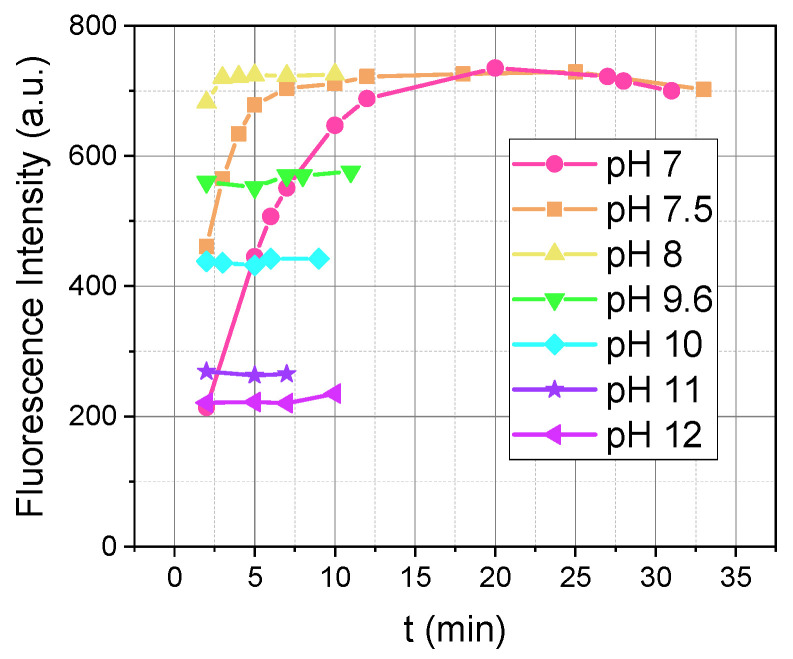
Influence of pH on the fluorescence intensity over time (λ_ex_/λ_em_ = 340/460 nm) during the reaction between 5 µM of dopamine and 500 µM of OPA with 2000 µM of MPTES, recorded at M 5/5.

The reaction product formed from dopamine, OPA, and MPTES exhibits fluorescence. The excitation wavelength was found at 340 nm, and the emission wavelength at 460 nm. The fluorescence intensity is dependent on the pH of the reaction medium. Figure 1 shows the comparison of the fluorescence intensity of the reaction product over time at various pHs. As we can see from Figure 1, the pH affects two parameters: fluorescence intensity and reaction rate.

In Figure 1, in the pH range of 7 to 8, fluorescence intensity reached the same maximum value but at different times: a pH of 7 after 20 min, a pH of 7.5 after 7 min, and a pH of 8 after 3 min. The reaction in this range exhibited a rapid initial increase in fluorescence, followed by stabilization at a high intensity.

For pH values between 8 and 12, the fluorescence intensity decreased as the pH increased (Figure 1). In [16], it was shown that at pH values above 8, dopamine oxidation occurs. It is possible that the oxidation reaction competes with OPA/MPTES, and as the pH increases, less dopamine is available to react with OPA/MPTES, resulting in decreased fluorescence intensity.

Based on these data, we suggest that a pH of 8 is the most optimal condition due to its high fluorescence intensity and the rapid achievement of the maximum signal (Figure 1).

### 3.2. Influence of Dopamine-to-OPA Ratio on Fluorescence Intensity

**Figure 2:**
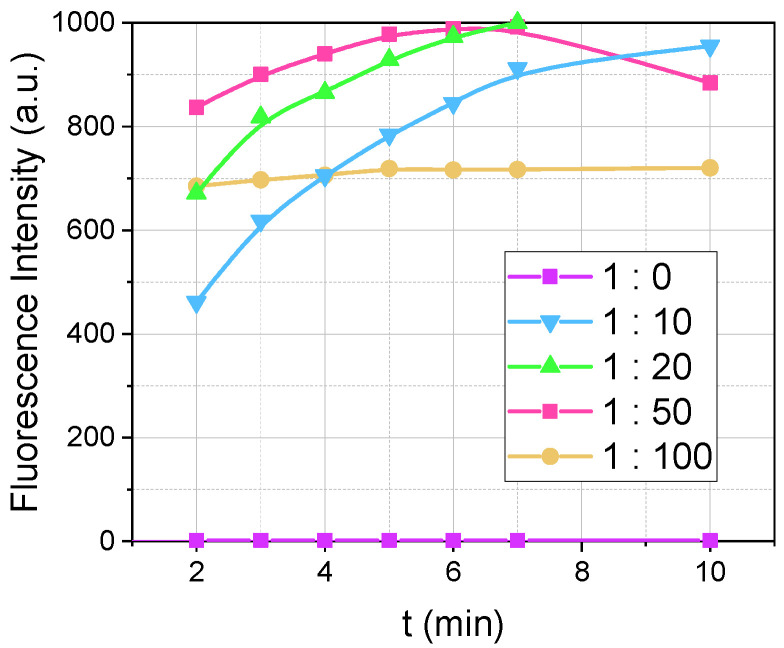
Influence of the dopamine-to-OPA ratio on the fluorescence intensity over time (λ_ex_/λ_em_ = 340/460 nm) during the reaction between 5 µM of dopamine and OPA (0–500 µM) with MPTES (OPA:MPTES = 1:4) at a pH of 8, recorded at M 5/5. The legend indicates the ratio of dopamine to OPA as follows: 1:0 (5 µM:0 µM), 1:10 (5 µM:50 µM), 1:20 (5 µM:100 µM), 1:50 (5 µM:250 µM), and 1:100 (5 µM:500 µM).

We investigated how varying the ratio of dopamine to OPA affects the fluorescence intensity, as illustrated in Figure 2. The dopamine concentration was fixed at 5 µM, while the concentrations of OPA varied: 0 µM, 50 µM, 100 µM, 250 µM, and 500 µM, corresponding to dopamine: OPA ratios of 1:0, 1:10, 1:20, 1:50, and 1:100, respectively. In experiments with OPA (such as those at 1:10 and higher), the MPTES was added at a fourfold excess relative to OPA (OPA:MPTES = 1:4). Consequently, for the 1:10 ratio, the reaction mixture contained 5 µM of dopamine, 50 µM of OPA, and 200 µM of MPTES, while for the 1:100 ratio, it included 5 µM of dopamine, 500 µM of OPA, and 2 mM of MPTES.

In the absence of OPA and MPTES (ratio 1:0), fluorescence was significantly weaker, manifesting at λ_ex_/λ_em_ = 280/320 nm, which lies outside the main emission range we measured for the fluorescent product formed by dopamine, OPA, and MPTES (340/460 nm). As the OPA concentration increased from 50 µM to 250 µM, not only did the fluorescence intensity increase but the reaction also proceeded at a faster rate. However, at an OPA concentration of 500 µM (ratio 1:100), we observed partial fluorescence quenching. This quenching at higher OPA concentrations can be attributed to the significant presence of MPTES, leading to opalescence in the reaction medium due to the condensation reaction of MPTES. This opalescence is hypothesized to be the reason why the maximum fluorescence intensity at 500 µM of OPA reaches only 700 a.u., compared to 1000 a.u. observed at OPA concentrations ranging from 50 µM to 250 µM. Therefore, OPA concentrations ranging from 50 µM to 250 µM appear to be suitable for method development, with 100 µM being identified as the most optimal to ensure a rapid reaction rate without the effects of quenching.

### 3.3. Influence of OPA-to-MPTES Ratio on Reaction Efficiency

**Figure 3:**
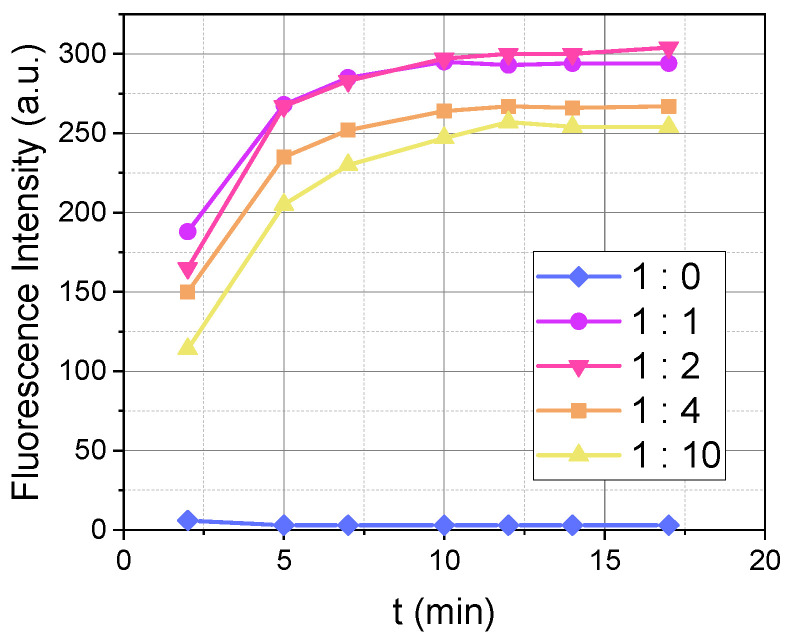
Influence of the OPA-to-MPTES ratio on the fluorescence intensity over time (λ_ex_/λ_em_ = 340/460 nm) during the reaction between 5 µM of dopamine and 100 µM of OPA with MPTES (0–1000 µM) at a pH of 8, recorded at M 3/3. The legend indicates the ratio of OPA to MPTES as follows: 1:0 (100 µM:0 µM), 1:1 (100 µM:100 µM), 1:2 (100 µM:200 µM), 1:4 (100 µM:400 µM), and 1:10 (100 µM:1000 µM).

We investigated the influence of the different ratios of OPA to MPTES on the efficiency of the reaction with dopamine (Figure 3). Dopamine and OPA concentrations were fixed at 5 µM and 100 µM, respectively. The OPA:MPTES ratios tested were 1:0, 1:1, 1:2, 1:4, and 1:10.

The experimental results (Figure 3) showed that the presence of MPTES in the reaction medium significantly increases the fluorescence intensity of the reaction between dopamine and OPA, which is practically zero at medium lamp power in the absence of MPTES (ratio 1:0).

As previously observed, pH and OPA concentrations influence the reaction rate. In contrast, the concentration of MPTES had no noticeable effect on the reaction kinetics. As shown in Figure 3, the time-dependent fluorescence intensity profiles for different MPTES concentrations are nearly identical, indicating that MPTES does not impact the rate of the reaction.

Ratio 1:1 is sufficient to facilitate the reaction between dopamine, OPA, and MPTES. However, an excess of the second thiol nucleophile ensures the complete progression of the reaction, enhancing the overall efficiency.

### 3.4. Fluorescent Properties of the Reaction Under Optimized Conditions

**Figure 4:**
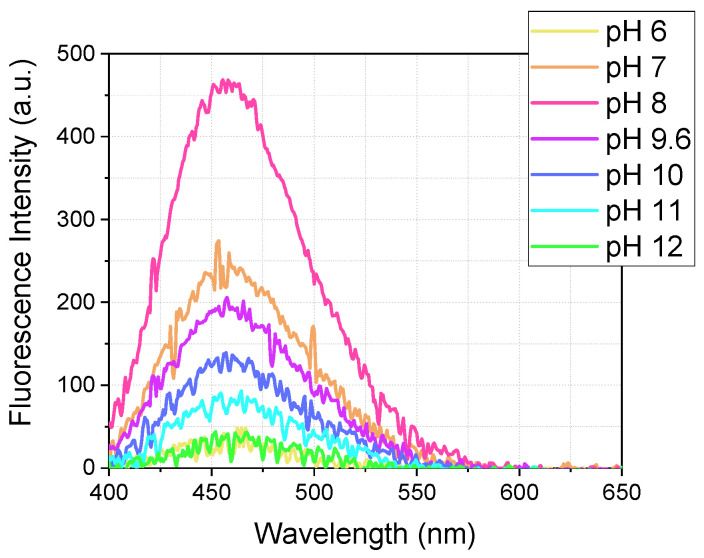
Fluorescence spectra (λ_ex_ = 340 nm) of the reaction between 5 µM of dopamine and 100 µM of OPA with 200 µM of MPTES at different pHs, recorded at M 3/3 after 10 min.

It has previously been shown that pH is a key factor influencing the fluorescence signal, as it determines both the intensity and the rate of the reaction. Further experiments revealed that different concentrations of OPA and MPTES are more suitable for this reaction. Therefore, it was of interest to investigate how these concentration changes would affect the pH dependence of fluorescence. The results of the pH influence on the fluorescence of the dopamine reaction with OPA and MPTES at optimized concentrations are presented in Figure 4.

To investigate the effect of pH on fluorescence intensity and emission wavelength, a series of experiments was conducted with pH values ranging from 6 to 12. The dopamine concentration was set at 5 µM, while the OPA concentration was 100 µM, and MPTES was 200 µM. The reaction was carried out for 10 min at a constant temperature of 25 °C. The excitation wavelength was fixed at 340 nm. The excitation and emission slit was 3 nm. The data obtained (Figure 4) demonstrated that the pH significantly influences the fluorescence intensity of the reaction product without shifts; the emission wavelength was 460 nm. Maximum intensity was observed at a pH of 8, indicating optimal reaction conditions for fluorophore formation.

**Figure 5:**
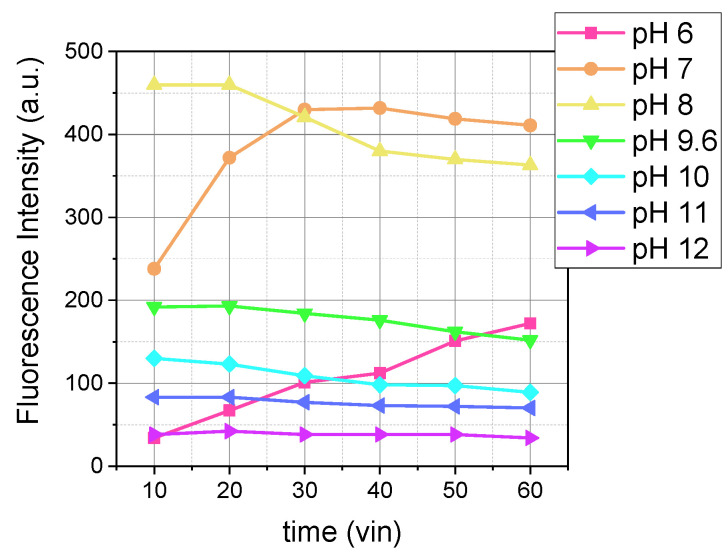
Influence of pH on the fluorescence intensity over time (λ_ex_/λ_em_ = 340/460 nm) during the reaction between 5 µM of dopamine and 100 µM of OPA with 200 µM of MPTES, recorded at M 3/3.

In addition to recording spectra at different pH values, the behavior of the fluorescence intensity of the reaction product over time was also studied (Figure 5). After optimizing the concentrations of OPA and MPTES, the general pattern of pH influence on intensity remained consistent, as seen when comparing Figure 5 with Figure 1. At a pH of 7–8, the intensity is higher than at a pH of 9.6–12. As the pH increases from 8 to 12, a decrease in intensity is observed. Furthermore, as in Figure 1, the intensity values at a pH of 9.6–12 remain relatively stable.

Interestingly, the optimization of OPA and MPTES concentrations affected the reaction kinetics at a pH of 7. At higher concentrations of OPA and MPTES (500 µM of OPA and 2000 µM of MPTES), the maximum intensity at a pH of 7 was reached at the 20th minute after the start of the reaction (Figure 1), whereas at 100 µM of OPA and 200 µM of MPTES, the maximum intensity was reached at the 30th minute (Figure 5). This indicates that at a pH of 7, the reaction rate is more sensitive to the indicator concentration than at a pH of 8–12.

**Figure 6:**
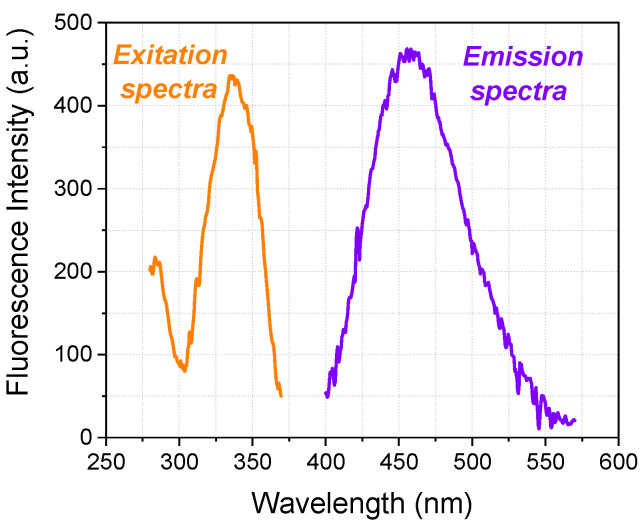
Fluorescence spectra (λ_ex_/λ_em_ = 340/460 nm) of the reaction between 5 µM of dopamine and 100 µM of OPA with 200 µM of MPTES at a pH of 8, recorded at M 3/3 after 10 min.

The excitation spectrum of the reaction product has a maximum at 340 nm, while the emission spectrum peaks at 460 nm, resulting in a Stokes shift of 120 nm (Figure 6). According to the literature, fluorophore dyes such as fluorescein, rhodamine, and cyanine exhibit small Stokes shifts, ranging from 13 to 36 nm [23]. Furthermore, reference [23] notes that Stokes shifts greater than 80 nm are considered large and can minimize cross-talk between the excitation source and fluorescent emission. As shown in Figure 6, the excitation and emission spectra for the reaction product of dopamine and OPA with MPTES are well separated without overlap.

### 3.5. The Linear Relationship Between Fluorescence Intensity and Dopamine Concentration

**Figure 7:**
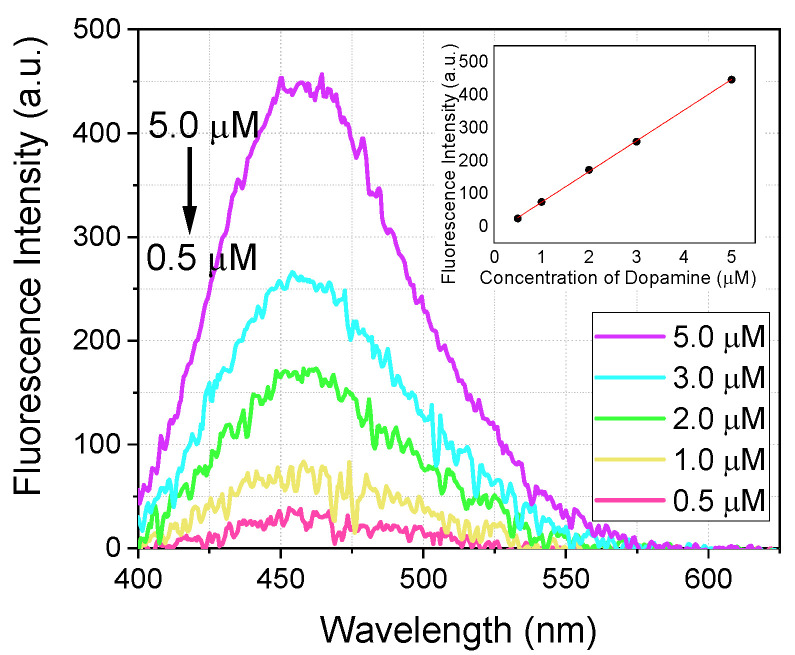
Fluorescence spectra and linear calibration plot (λ_ex_/λ_em_ = 340/460 nm) of the reaction between dopamine (0.5, 1.0, 2.0, 3.0, and 5.0 µM) and 100 µM of OPA with 200 µM of MPTES at a pH of 8, recorded at M 3/3 after 10 min.

A verification of the linear relationship between fluorescence intensity and dopamine concentration was conducted under conditions λ_ex_/λ_em_ = 340/460 nm, a concentration range of dopamine 0.5–5 µM, 100 µM of OPA with 200 µM of MPTES at a pH of 8. The fluorescence spectra at various dopamine concentrations, along with the resulting calibration curve, are presented in Figure 7. The concentration range of 0.5–5.0 µM of dopamine has a linear relationship with a high adjusted R-squared (Adj.R^2^ is 0.999). The calculated LOD was 130 nM.

### 3.6. Effect of Fluorimeter Parameter on Detection Sensitivity

**Figure 8:**
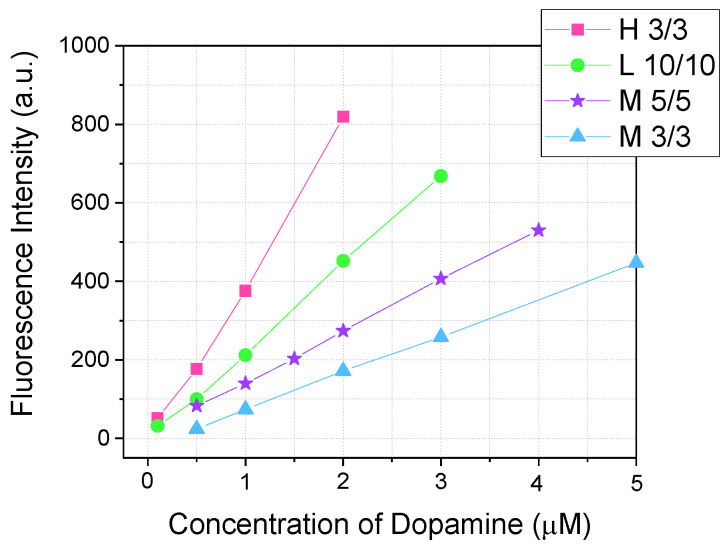
Calibration plots (λ_ex_/λ_em_ = 340/460 nm) of the reaction between dopamine and 100 µM of OPA with 200 µM of MPTES at a pH of 8, recorded after 10 min.

We measured calibration solutions of dopamine after reaction with OPA and MPTES at the 10th minute, using different lamp power settings of the fluorimeter. The slit sizes for excitation and emission were also varied. The spectral scan rate was constant at 500 nm/min. Calibration plots for different parameters are shown in Figure 8.

A comparison of high and medium lamp power at 3/3 nm slits showed that the slope of the calibration curve differs (Table 2). For medium power, the slope was 9.3 × 10^7^, while for high power, it was almost four times higher—4.09 × 10^8^. This indicates that higher lamp power provides a more sensitive system. As shown in Table 2, the LOD for a 3/3 nm high power is 46.8 nM, whereas for a 3/3 nm medium power, it is 130 nM.

An increase in slit size resulted in a higher slope of the calibration curve: for a 3/3 nm medium power, the slope was 9.3 × 10^7^, compared to 1.34 × 10^8^ for 5/5 nm (Table 2). This is due to the wider slits allowing more photons to pass through, thereby increasing the fluorescence intensity. Additionally, increasing the slit size reduces noise by decreasing fluctuations in photon flux. This is evident from the standard deviation (SD) of the blank solution: for a 3/3 nm medium power, the SD was 4.10, while for a 5/5 nm, it was 1.46 (Table 2).

Among the tested fluorimeter parameters, the highest accuracy was shown by using low lamp power with slit sizes of 10/10 nm (Table 2). After optimizing the parameters (lamp power and slit size), the limit of detection decreased from 130 nM to 8.7 nM, which corresponds to an improvement in sensitivity by more than one order of magnitude (Table 2).

### 3.7. Interference Study of the OPA/MPTES Detection System

**Figure 9:**
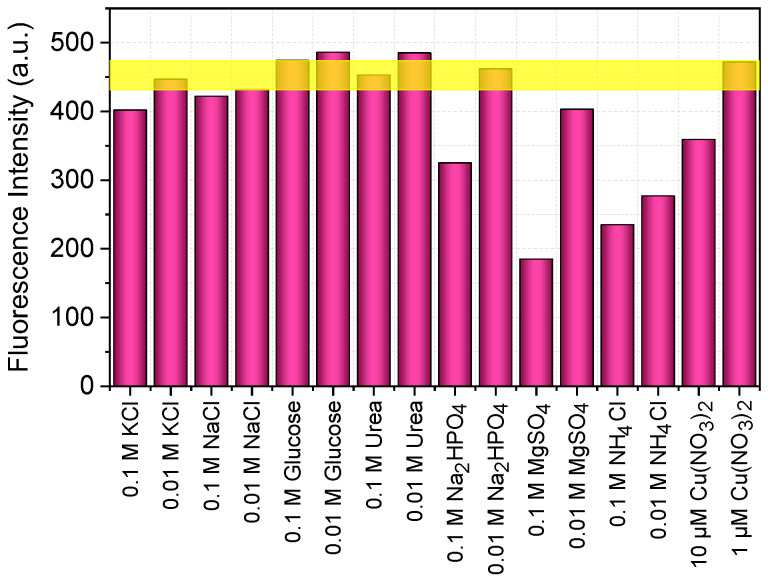
Fluorescence intensity (λ_ex_/λ_em_ = 340/460 nm) of the reaction between 5 µM of dopamine and 100 µM of OPA with 200 µM of MPTES at a pH of 8 in the presence of various solutes, recorded at M 3/3 after 10 min. The horizontal yellow band indicates the intensity range for the solution without added solutes.

To evaluate the influence of potential interferences on the resulting fluorescence, several soluble compounds were tested by introducing them prior to the reaction between dopamine and OPA with MPTES. Figure 9 presents a diagram illustrating the fluorescence intensity in the presence of potassium chloride, sodium chloride, glucose, urea, sodium dihydrogen phosphate, magnesium sulfate, and ammonium chloride, each tested at concentrations of 0.1 and 0.01 M. Additionally, copper (II) nitrate was tested at concentrations of 1 and 10 µM. The fluorescence intensity range of dopamine in the absence of interferences is indicated by the yellow horizontal line.

The presence of glucose, urea, sodium chloride, and potassium chloride in the reaction medium has almost no effect on signal intensity (Figure 9). Sodium dihydrogen phosphate at a concentration of 0.01 M also has no influence, but at 0.1 M, it reduces the intensity by 25%, which is attributed to an increase in the pH of the reaction medium (Figure 9). Magnesium sulfate has a more pronounced effect: at a concentration of 0.01 M, the intensity decreases by more than 10%, and at 0.1 M, by more than 50% (Figure 9).

Copper ions are known to form complexes with dopamine, which likely interferes with the reaction between dopamine and OPA with MPTES. At a copper concentration of 1 µM, there is no effect on fluorescence intensity, and the signal remains within the range typical for dopamine without interferences (Figure 9). However, increasing the copper concentration to 10 µM reduces fluorescence intensity by 10% (Figure 9). Ammonium ions have a strong effect on the reaction, making their use in this method unacceptable (Figure 9).

### 3.8. Selectivity Study of the OPA/MPTES Detection System for Various Biogenic Amines

The selectivity of the OPA/MPTES detection system was evaluated under the optimal conditions established for dopamine, to assess its applicability for detecting dopamine in the presence of other biogenic amines. The system was tested using the same concentration of biogenic amines as for dopamine—5 µM, with OPA at a concentration of 100 µM and MPTES at 200 µM. The reaction was carried out at pHs of 6–12 for 10 min, and the excitation wavelength was set to 340 nm.

**Figure 10:**
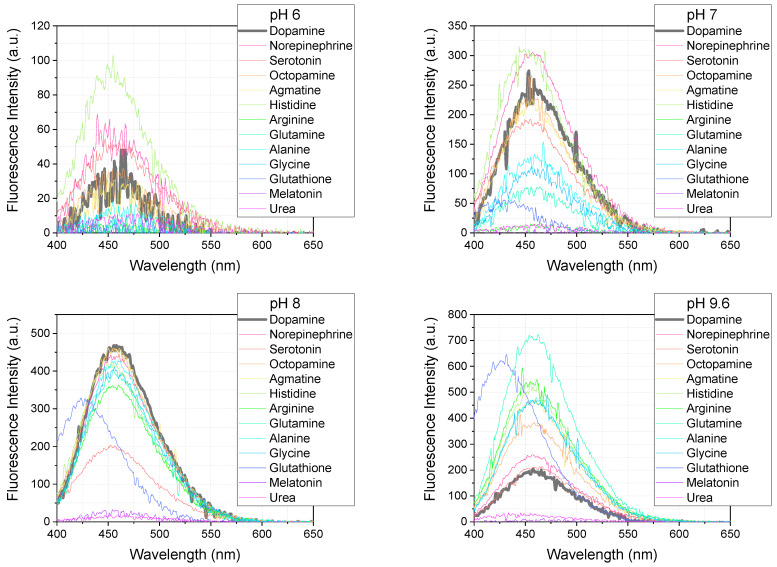

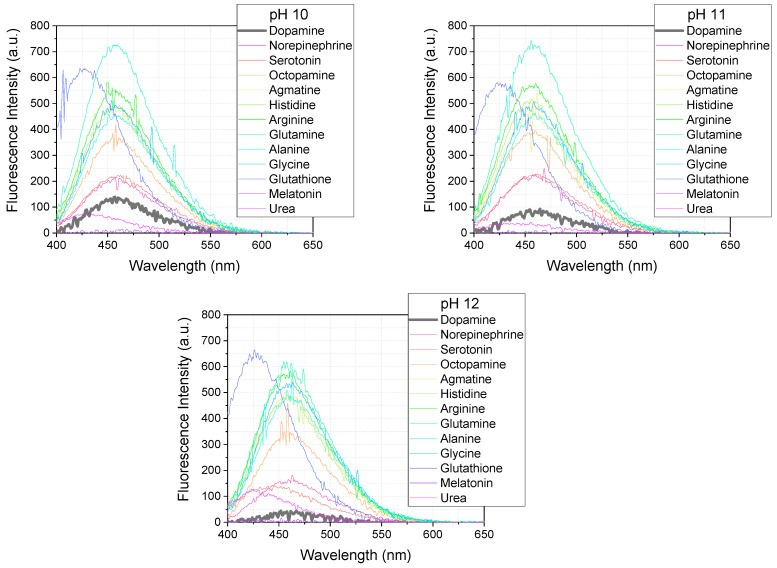
Fluorescence spectra (λ_ex_ = 340 nm) of the reaction between various biogenic amines (5 µM each) and 100 µM of OPA with 200 µM of MPTES, recorded at M 3/3 after 10 min.

Figure 10 shows the fluorescence spectra of the reaction products obtained under these conditions. Most of the biogenic amines, including dopamine, norepinephrine, serotonin, octopamine, agmatine, histidine, arginine, glutamine, alanine, and glycine, exhibit an emission maximum at 450–460 nm. Interestingly, glutathione shows a distinct emission maximum at 426 nm, while the signal intensity for urea and melatonin is very low.

**Figure 11:**
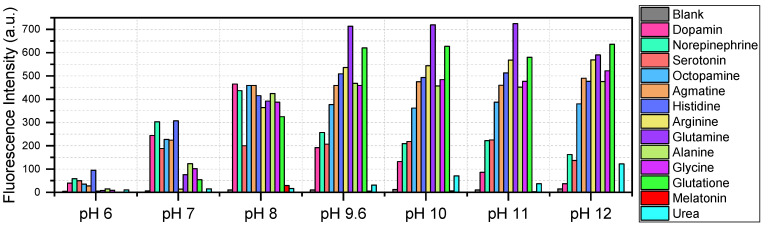
Fluorescence intensity (λ_ex_/λ_em_ = 340/460 nm, exept for glutation λ_em_ = 426 nm) of the reaction between various biogenic amines (5 µM each) and 100 µM of OPA with 200 µM of MPTES, recorded at M 3/3 after 10 min.

Although the method lacks inherent selectivity for dopamine, the obtained results (Figure 11) demonstrate that the OPA/MPTES system can be applied to the detection of a wide range of biologically significant amines. This expands its potential use in analytical applications, particularly when combined with prior separation techniques [24].

Moreover, the preliminary removal of amino acids from urine samples can improve the accuracy of dopamine analysis and make this approach applicable to real samples. At the same time, the developed method can be used to evaluate the sorption properties of various sorbents with respect to amino acids, provided that additional studies are conducted and calibration for individual amino acids is performed. Mesoporous silicas with functional groups capable of interacting with the carboxyl group of amino acids [25,26] should be considered as potential sorbents. Mesoporous silicas possess high sorption capacity and can be easily separated using a syringe filter. These directions are of interest for further research, which could expand the application of the developed method not only in dopamine analysis in real samples but also in studying the use of nanomaterials in biologically significant systems. 

Figure 11 presents a fluorescence intensity diagram derived from the spectra, providing an overview of their relative relationships. As seen from the diagram, at a pH of 8, other amines do not exceed the intensity of the signal from the reaction product with dopamine.

## 4. Conclusions

In this study, we systematically investigated the conditions for optimizing the fluorescent determination of dopamine using a reaction with o-phthalaldehyde (OPA) and 3-mercaptopropyltriethoxysilane (MPTES). It was found that the optimal conditions for achieving the highest fluorescence signal include a pH of 8, an OPA concentration of 100 µM, and an MPTES concentration of 200 µM, with a reaction time of 10 min.

This study demonstrated that spectrofluorimeter settings, such as the width of excitation and emission slits as well as lamp power, significantly affect the sensitivity of the method. Transitioning from medium power with a slit width of 3 nm to low power with a slit width of 10 nm allowed for a reduction in background noise and an increase in fluorescence intensity, leading to a decrease in the detection limit from 130 nM to 8.7 nM.

The stability of the fluorescence signal in the presence of potential interferents was also examined. High concentrations (0.1 M) of NaCl, glucose, and urea did not noticeably affect the signal, whereas MgSO_4_ and NaH_2_PO_4_ reduced fluorescence intensity due to their impact on pH.

The proposed approach is rapid, single-step, and requires no additional sample processing, making it promising for analytical applications. Although OPA reacts not only with dopamine but also with other amino acids, this broad specificity expands the method’s utility for amino acid detection. Moreover, incorporating preliminary separation techniques (e.g., extraction or sorption) can render the assay more selective for dopamine in complex biological matrices.

Finally, these findings may foster the development of portable fluorimeters with a limited set of excitation/emission wavelengths, offering a cost-effective means of detecting dopamine and amino acids at microconcentration levels. Such an approach could significantly broaden the accessibility of fluorescence-based analyses in diverse laboratory settings.

## Figures and Tables

**Table 1:** The comparison of the spectrofluorescence methods for dopamine detection.

Reagents	λ_ex_/λ_em_, nm	LOD	[Ref.]
Direct measurements in methanol	279/315	0.082 μg/mL (500 nM)	[16]
Oxidation product of the dopamine with NaOH to obtain Polydopamine	370/470–530	40 nM	[17]
Alkali, polyethylene-imine to obtain Polydopamine	370/526	0.3 μM (300 nM)	[18]
Mercury (II) and ethylene diamine	393/447	18 ng/mL (117 nM)	[19]
Chlorosulfonyl-thenoyltrifluoro-acetone Europium Probe	385/612	32 nM	[20]
3-Hydroxyphenyl-boronic acid	417/470	20 nM	[21]
Resorcinol	420/475	1.8 nM	[22]
OPA/Pluronic F127	390/455	11 nM	[5]
OPA/MPTES	340/460	8.7 nM	this study

**Table 2:** Linear parameters calculated from calibration curves obtained from fluorescence spectra under various lamp power settings of the fluorimeter and different excitation/emission slit widths.

Lamp power and ex/em Slits	High 3 nm/3 nm	Medium 3 nm/3 nm	Medium 5 nm/5 nm	Low 10 nm/10 nm
concentration range of dopamine, µM	0.1–2.0	0.5–5.0	0.5–4.0	0.1–3.0
adj. R^2^	0.994	0.999	0.995	0.998
slope	4.09 × 10^8^	9.3 × 10^7^	1.34 × 10^8^	2.2 × 10^8^
SD of blank	6.38	4.10	1.46	0.65
LOD, nM	46.8	130	32.7	8.7
LOQ, nM	156	440	109	29

## Data Availability

The original contributions presented in this study are included in the article. Further inquiries can be directed to the corresponding authors.
